# Supplementary material for: HIVprotI: an integrated web based platform for prediction and design of HIV proteins inhibitors
Source: J Cheminform. 2018 Mar 9;10:12. doi: 10.1186/s13321-018-0266-y (PMC5845081; doi:10.1186/s13321-018-0266-y)
Supplement: Supplementary file 2 — Additional file 2. Source code of HIVProtI web server. [file 13321_2018_266_MOESM2_ESM.zip › HIVprotI_Source-code/hivprotI/weka-3-6-6/doc/weka/filters/unsupervised/attribute/TimeSeriesTranslate.html]

TimeSeriesTranslate


---


|  |  |  |
| --- | --- | --- |
| **Overview** \| **Package** \| **Class** \| **Tree** \| **Deprecated** \| **Index** \| **Help** \| **Weka's home** | |  |
| **PREV CLASS**   **NEXT CLASS** | **FRAMES**    **NO FRAMES**     **All Classes** |  |
| SUMMARY: NESTED \| FIELD \| CONSTR \| METHOD | DETAIL: FIELD \| CONSTR \| METHOD |  |


---


## weka.filters.unsupervised.attribute Class TimeSeriesTranslate

```
java.lang.Object
  weka.filters.Filter
      weka.filters.unsupervised.attribute.AbstractTimeSeries
          weka.filters.unsupervised.attribute.TimeSeriesTranslate
```

**All Implemented Interfaces:**: java.io.Serializable, CapabilitiesHandler, OptionHandler, RevisionHandler, UnsupervisedFilter

**Direct Known Subclasses:**: TimeSeriesDelta

---

``` public class TimeSeriesTranslate extends AbstractTimeSeries ```

An instance filter that assumes instances form time-series data and replaces attribute values in the current instance with the equivalent attribute values of some previous (or future) instance. For instances where the desired value is unknown either the instance may be dropped, or missing values used. Skips the class attribute if it is set.


Valid options are:

```
 -R <index1,index2-index4,...>
  Specify list of columns to translate in time. First and
  last are valid indexes. (default none)
```

```
 -V
  Invert matching sense (i.e. calculate for all non-specified columns)
```

```
 -I <num>
  The number of instances forward to translate values
  between. A negative number indicates taking values from
  a past instance. (default -1)
```

```
 -M
  For instances at the beginning or end of the dataset where
  the translated values are not known, remove those instances
  (default is to use missing values).
```

**Version:**
:   $Revision: 5547 $

**Author:**
:   Len Trigg (trigg@cs.waikato.ac.nz)

**See Also:**: Serialized Form

---

**Constructor Summary**

`TimeSeriesTranslate()`


| **Method Summary** | |
| --- | --- |
| `Capabilities` | `getCapabilities()`             Returns the Capabilities of this filter. |
| `java.lang.String` | `getRevision()`             Returns the revision string. |
| `java.lang.String` | `globalInfo()`             Returns a string describing this classifier |
| `static void` | `main(java.lang.String[] argv)`             Main method for testing this class. |
| `boolean` | `setInputFormat(Instances instanceInfo)`             Sets the format of the input instances. |

**Methods inherited from class weka.filters.unsupervised.attribute.AbstractTimeSeries**

`attributeIndicesTipText, batchFinished, fillWithMissingTipText, getAttributeIndices, getFillWithMissing, getInstanceRange, getInvertSelection, getOptions, input, instanceRangeTipText, invertSelectionTipText, listOptions, setAttributeIndices, setAttributeIndicesArray, setFillWithMissing, setInstanceRange, setInvertSelection, setOptions`

**Methods inherited from class weka.filters.Filter**

`batchFilterFile, filterFile, getCapabilities, getOutputFormat, isFirstBatchDone, isNewBatch, isOutputFormatDefined, makeCopies, makeCopy, numPendingOutput, output, outputPeek, toString, useFilter, wekaStaticWrapper`

**Methods inherited from class java.lang.Object**

`equals, getClass, hashCode, notify, notifyAll, wait, wait, wait`

**Constructor Detail**

### TimeSeriesTranslate

```
public TimeSeriesTranslate()
```


**Method Detail**

### globalInfo

```
public java.lang.String globalInfo()
```

:   Returns a string describing this classifier

    :   **Returns:**: a description of the classifier suitable for displaying in the explorer/experimenter gui

---


### getCapabilities

```
public Capabilities getCapabilities()
```

:   Returns the Capabilities of this filter.

    :   **Specified by:**: `getCapabilities` in interface `CapabilitiesHandler` **Overrides:**: `getCapabilities` in class `Filter`
    :   **Returns:**: the capabilities of this object **See Also:**: `Capabilities`

---


### setInputFormat

```
public boolean setInputFormat(Instances instanceInfo)
                       throws java.lang.Exception
```

:   Sets the format of the input instances.

    :   **Overrides:**: `setInputFormat` in class `AbstractTimeSeries`
    :   **Parameters:**: `instanceInfo` - an Instances object containing the input instance structure (any instances contained in the object are ignored - only the structure is required). **Returns:**: true if the outputFormat may be collected immediately **Throws:**: `UnsupportedAttributeTypeException` - if selected attributes are not numeric or nominal.: `java.lang.Exception` - if the format couldn't be set successfully

---


### getRevision

```
public java.lang.String getRevision()
```

:   Returns the revision string.

    :   **Specified by:**: `getRevision` in interface `RevisionHandler` **Overrides:**: `getRevision` in class `Filter`
    :   **Returns:**: the revision

---


### main

```
public static void main(java.lang.String[] argv)
```

:   Main method for testing this class.

    :   **Parameters:**: `argv` - should contain arguments to the filter: use -h for help


---


|  |  |  |
| --- | --- | --- |
| **Overview** \| **Package** \| **Class** \| **Tree** \| **Deprecated** \| **Index** \| **Help** \| **Weka's home** | |  |
| **PREV CLASS**   **NEXT CLASS** | **FRAMES**    **NO FRAMES**     **All Classes** |  |
| SUMMARY: NESTED \| FIELD \| CONSTR \| METHOD | DETAIL: FIELD \| CONSTR \| METHOD |  |


---
